# Gastric Volvulus, An Important Yet Commonly Overlooked Etiology of Upper Gastrointestinal Bleeding: A Case Study

**DOI:** 10.7759/cureus.26976

**Published:** 2022-07-18

**Authors:** Yasir Rajwana, Kosisochukwu J Ezeh, William Ott, Etan Spira

**Affiliations:** 1 Internal Medicine, Jersey City Medical Center, Jersey City, USA; 2 Gastroenterology and Hepatology, Jersey City Medical Center, Jersey City, USA

**Keywords:** vascular compromise, paraesophageal hiatal hernia, gastrectomy, acute gastrointestinal bleed, gastric volvulus

## Abstract

Gastric volvulus is a distinct and uncommon pathology that usually presents with vomiting secondary to gastric outlet obstruction and gastrointestinal bleeding with an association with hiatal hernia. We present a case of a 71-year-old female who presented to the emergency department (ED) with a three-day history of coffee ground emesis. Of note, the patient was recently in the hospital under medical observation two weeks prior, with similar complaints of hematemesis. Chest X-ray revealed a left basilar opacity representing bowel gas suggestive of a hiatal hernia. Intravenous proton pump inhibitors were initiated but due to persistent recurrence of symptoms and progressive discomfort, a computed tomography (CT) of the chest and abdomen was ordered. This revealed a partial gastric volvulus with signs suggestive of vascular compromise of the herniated part of the stomach. She subsequently underwent emergent laparotomy, repair of the hiatal hernia, and partial gastrectomy and gastropexy. Post-surgical biopsy findings showed focal mucosal necrosis and ulceration, focal foveolar hyperplasia, edematous changes, and overall congestion in the submucosal tissue. She was discharged five days later with no complications or recurrence of symptoms.

## Introduction

Gastric volvulus is the twisting of all or a portion of the stomach by at least 180 degrees, resulting in a closed-loop obstruction that can result in complications, including but not limited to gastrointestinal (GI) bleeding [1]. We report a case of a gastric volvulus associated with a large hiatal hernia as a rare etiology of upper GI bleeding. According to a classification proposed by Singleton based on the axis around which the stomach rotates, there are three main kinds: organoaxial, mesenteroaxial, and combined [2].

In an organoaxial gastric volvulus, the esophagogastric junction (EGJ) and the pylorus form an axis around which the stomach spins. The antrum revolves in the opposite direction from the stomach's fundus. In around 59% of instances [3], this kind of gastric volvulus occurs, and it is typically accompanied by diaphragmatic abnormalities. Organoaxial gastric volvulus frequently results in strangulation and necrosis, which have been documented in 5-28 % of cases [4], whereas in the mesenteroaxial type, the lesser and bigger curvatures are divided by the mesenteroaxial axis. The posterior surface of the stomach lies anteriorly as a result of the antrum rotating anteriorly and superiorly. Typically, the rotation is sporadic and incomplete. It is rare to have vascular compromise [2].

Hiatal hernias, a type of hernia in which abdominal organs (most often the stomach) migrate through the diaphragm into the middle compartment of the thoracic cavity, are an issue whose incidence increases with age. Approximately 60% of individuals aged 50 or older have a hiatal hernia [5]. Hiatal hernias themselves are largely either defined as sliding or para-esophageal. Type I, or sliding hernia, is referred to as a displacement at the gastroesophageal (GE) junction above the diaphragm. The stomach and, more specifically, the fundus remain below the GE junction. Types II, III, and IV are all para-esophageal hernias, which are true hernias and characterized by an upward dislocation of the gastric fundus through a defect in the phrenoesophageal membrane [6].

This article was previously presented as a meeting abstract at the 2021 ACG Annual Scientific Meeting on October 27, 2021 [1].

## Case presentation

We present a case of a 71-year-old female with a history of hypertension, chronic alcohol abuse (about three beers every other day) with associated gastritis, and a recent upper GI bleed who presented to the emergency department with a three-day history of coffee ground emesis. She reported taking non-steroidal anti-inflammatory drugs (NSAIDs) occasionally for aches and pains about once or twice per week. She denied any difficulty breathing, abdominal pain, weight loss, melena, altered consciousness, or a history of liver disease.

The patient was recently in the hospital under medical observation two weeks prior with similar complaints of hematemesis. At the time, she had remained hemodynamically stable with no concerns over hypovolemia and her hemoglobin remained greater than 13, which was at her baseline. She ultimately would undergo an endoscopy that revealed a medium hiatal hernia without obstruction in the distal esophagus, acute moderate diffuse superficial gastritis of the antrum, and mild diffuse superficial duodenitis. The patient was discharged with instructions to continue taking pantoprazole daily and to follow up as an outpatient in two weeks as well as at that time obtaining a CT of her abdomen. She admits she was non-compliant with any of these requests.

While in the emergency department during this current presentation, her blood pressure was 175/89 mmHg, heart rate at 64 beats per minute, respirations unlabored at 18 breaths per minute while saturating 95% on room air, and afebrile at 98.0 F. On physical exam, she was found to be in mild distress but fully oriented, with no mucosal pallor, no cardiac murmurs, and no significant abdominal tenderness nor rebound as well as no abdominal. In addition, there was no palmar erythema, Dupuytren's contracture, or spider nevi. Laboratory findings showed hemoglobin of 16 g/dl, platelets of 233 k/uL, prothrombin time (PT) of 13.1s, international normalized ratio (INR) of 0.95, blood urea nitrogen (BUN) of 13, creatinine of 0.80, albumin of 5.1 g/dL, alanine aminotransferase (ALT) 24 Unit/L, aspartate aminotransferase (AST) of 30 Unit/L, alkaline phosphatase (ALP) of 116 Unit/L, Lactic acid 1.6 mmol/L, bicarbonate 34 mmol/L, and serum sodium 146 mmol/L.

**Figure 1:**
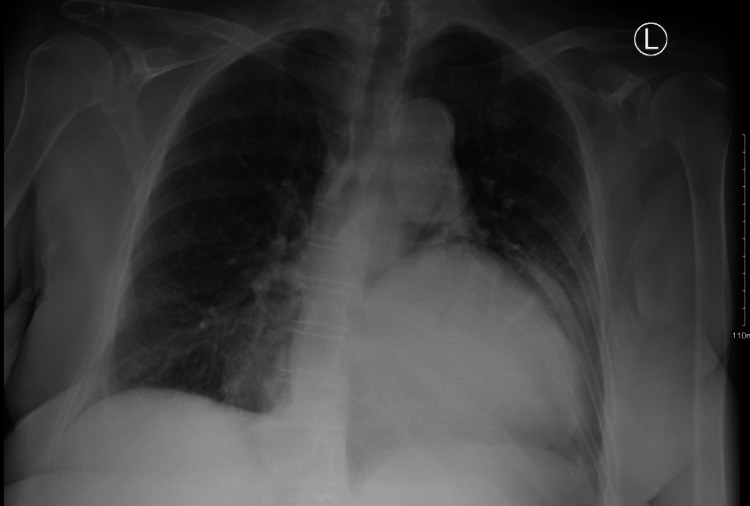
Radiograph chest posteroanterior (PA) view shows a left basilar opacity representing bowel gas suggestive of a hiatal hernia

Chest X-ray, as seen in Figure 1, revealed a left basilar opacity representing bowel gas suggestive of hiatal hernia.

**Figure 2:**
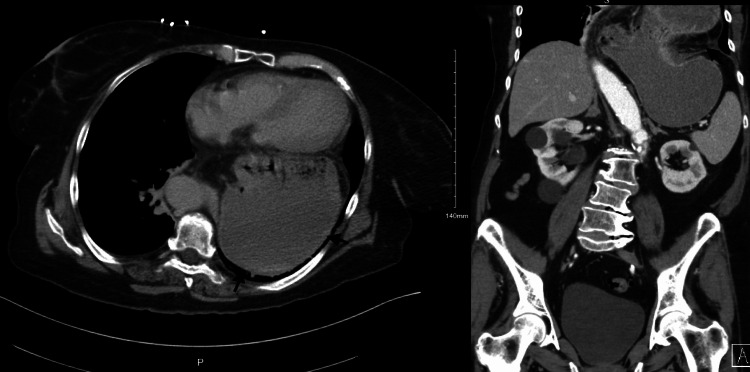
Left: Axial CT image shows the retrocardiac position of the stomach. Right: Coronal reformatted CT images show a large diaphragmatic defect with portions of the stomach within the thoracic cavity

Subsequently, intravenous proton pump inhibitors were initiated. Ultimately, due to her recurrent presenting symptoms and level of progressive discomfort, CT of the chest and abdomen were ordered, illuminating partial volvulus with signs suggestive of vascular compromise of the herniated part of the stomach as seen in Figure 2.

She underwent emergent laparotomy, repair of the hiatal hernia, and partial gastrectomy and gastropexy. Post-surgical histopathological findings would show focal mucosal necrosis/ulceration, focal foveolar hyperplasia, edematous changes, and overall congestion in the submucosal tissue. Eventually, she was discharged five days later with no complications or recurrence of symptoms.

## Discussion

Acute gastric volvulus is a distinct and uncommon pathology that usually presents with vomiting secondary to gastric outlet obstruction and GI bleeding related to mucosal vascular compromise and sloughing. It is imperative to recall the association of a para-esophageal hiatal hernia with gastric volvulus during GI bleed evaluation, as it can ultimately lead to incarceration and potentially fatal strangulation of vital organs.

Gastric volvulus in particular is characterized by rotation of the stomach along its long or short axis leading to variable degrees of gastric outlet obstruction, which may present acutely or chronically. Especially in chronic rotation of the stomach, decreased venous return can occur, which leads to increased capillary pressure owing to gastric bleeding as seen in this case. Mortality related to acute gastric volvulus is high if unrecognized, underscoring the need for early diagnosis and treatment [7]. The combination of pain, vomiting, and an inability to pass a nasogastric tube, known as Borchardt’s triad, is present in as many as 70% of patients with acute gastric volvulus [8].

The initial diagnostic test to evaluate for volvulus of the stomach is typically plain radiography, which illustrates a single, large, spherical gas bubble located in the upper abdomen or chest with an air-fluid level [9]. Sometimes though, classic features are not present on plain radiography in regards to acute gastric volvulus, which necessitates the use of CT as compared to gastrointestinal contrast studies.

As held true in this case, the initial plain film of the patient’s chest alluded to the diagnosis, but the extent of its consequence would need further elucidation with additional imaging modalities. Both types of images can show abnormal positioning of the stomach, but a CT scan offers additional visual insight into the relationship of the stomach to its surrounding structures. This can offer the added benefit of delineating anatomic abnormalities associated with secondary gastric volvulus. As evidence, in a study of 36 patients with acute gastric volvulus, barium swallow was diagnostic in only two of four patients, whereas CT scan diagnosed all 26 patients in which it was utilized [10].

Management and treatment may differ slightly whether primary or secondary gastric volvulus is present but tend to both result in the need for gastric fixation or gastropexy after gastric reduction and de-rotation. If anatomical defects are present, it has been found to be prudent to repair these at the same time so as to avoid future recurrent volvulus. If tissue during this process is deemed to be nonviable then gastrectomy becomes warranted as well. Of note, poor surgical candidates have the alternative option of attempts at endoscopic de-rotation and gastric fixation with subsequent percutaneous endoscopic gastrostomy tube placement [11].

## Conclusions

Acute gastric volvulus is a distinct and uncommon pathology that usually presents with vomiting secondary to gastric outlet obstruction and gastrointestinal bleeding related to mucosal vascular compromise and sloughing. It is imperative for gastroenterologists to recall the association of para-esophageal hiatal hernia with gastric volvulus during gastrointestinal bleed evaluation, as it leads to incarceration and potentially fatal strangulation.
